# Doxorubicin Induces Bone Loss by Increasing Autophagy through a Mitochondrial ROS/TRPML1/TFEB Axis in Osteoclasts

**DOI:** 10.3390/antiox11081476

**Published:** 2022-07-28

**Authors:** Hyun-Jung Park, Sun-Young Yoon, Jung-Nam Park, Jae-Hee Suh, Hye-Seon Choi

**Affiliations:** 1 Department of Biological Sciences (BK21 Program), University of Ulsan, Ulsan 44610, Korea; fariypark@ulsan.ac.kr (H.-J.P.); tjsdud9981@mail.ulsan.ac.kr (S.-Y.Y.); elly1506@mail.ulsan.ac.kr (J.-N.P.); 2 Department of Pathology, Ulsan University Hospital, Ulsan 44030, Korea; drjhs@uuh.ulsan.kr

**Keywords:** autophagy, bone loss, doxorubicin, osteoclast, reactive oxygen species

## Abstract

Doxorubicin (DOX), a widely used chemotherapeutic agent, has been linked to an increased risk of bone damage in human patients and induces bone loss in mice. DOX induces autophagy, which contributes to bone homeostasis and excess autophagy in osteoclasts (OCs), resulting in bone loss. We hypothesized that DOX-induced bone loss is caused by the induction of autophagy in OCs. In vitro, DOX significantly increased the area of OCs and bone resorption activity, whereas it decreased OC number through apoptosis. DOX enhanced the level of LC3II and acidic vesicular organelles-containing cells in OCs, whereas an autophagy inhibitor, 3-methyladenine (3-MA), reversed these, indicating that enhanced autophagy was responsible for the effects of DOX. Increased mitochondrial reactive oxygen species (mROS) by DOX oxidized transient receptor potential mucolipin 1 (TRPML1) on the lysosomal membrane, which led to nuclear localization of transcription factor EB (TFEB), an autophagy-inducing transcription factor. In vivo, micro-computerized tomography analysis revealed that the injection of 3-MA reversed DOX-induced bone loss, and tartrate-resistant acid phosphatase staining showed that 3-MA reduced the area of OCs on the bone surface, which was enhanced upon DOX administration. Collectively, DOX-induced bone loss is at least partly attributable to autophagy upregulation in OCs via an mROS/TRPML1/TFEB axis.

## 1. Introduction

Autophagy is a homeostatic degradation mechanism that is closely associated with lysosomes. The cellular outcome of autophagy depends on its temporal nature and any imbalances in this process can result in negative outcomes [1]. Several studies have suggested that autophagy plays a critical role in bone metabolism. Pathway analyses in human genome-wide association studies have strongly associated the process of autophagy with osteoporosis [2]. The mutation of the autophagic cargo receptor SQSTM1 (p62) results in Paget’s disease of bone (PDB) by affecting the number of osteoclasts (OCs) and OC activity [3]. ATG5 and ATG7, both of which are autophagy-related proteins associated with autophagosome formation, have been found to play a critical role in OC regulation and bone density [4]. The down-regulation of MAILC3A, which plays an important role in autophagosome formation, has been shown to inhibit actin ring formation, thereby impairing resorption activity [5]. LPS-induced bone loss has been associated with enhanced autophagy in OCs [6,7]. An oxidized cholesterol metabolite, 7-ketocholesterol, also up-regulates RANKL-induced autophagy [8].

Doxorubicin (DOX), an anthracycline drug originally isolated from the fungus *Streptomyces peucetius* [9], is a potent anti-cancer drug. This compound has been clinically used to treat a variety of cancers, including breast cancer, bladder cancer, lymphoma, and acute lymphocytic leukemia [10]. DOX has been reported to inhibit topoisomerase II [11] and induces cell death by generating reactive oxygen species (ROS) [12]. Despite its efficacy as a chemotherapeutic agent, emerging evidence has demonstrated its side effects on bone. DOX-treated breast cancer patients exhibit low bone mineral density [13,14] and DOX-treated children also undergo long-term bone damage [15], suggesting that there is a strong association between DOX treatment and bone loss. Additionally, the induction of bone loss by DOX has also been confirmed in several animal models. DOX has been found to decrease trabecular bone volume and cortical bone thickness in rabbits [16], as well as mice and rats [17,18,19].

Recent work has suggested an involvement of autophagy in DOX resistance [20] and DOX-induced cardiotoxicity [21], suggesting that the effect of DOX on bone density may be mediated by autophagy. Therefore, we hypothesized that DOX enhances autophagy in OCs, resulting in bone loss. Additionally, we investigated the mechanisms through which DOX affects OCs and induces bone loss in mice.

## 2. Materials and Methods

### 2.1. Ethics Statement

All mice were kept separately in a pathogen-free facility. All animal care and procedures were performed in accordance with protocols and guidelines approved by the Ulsan University Animal Care and Use Committee (UOUACUC). The standards employed were also approved by the UOUACUC (HSC-21-030).

### 2.2. Reagents

Recombinant mouse M-CSF, RANKL, and antibodies against MCP-1 for coating and detection were obtained from R & D Systems, Inc. (Minneapolis, MN, USA). The assay kits for the RatLaps enzyme immunoassay (EIA), osteocalcin EIA, alkaline phosphatase (ALP), and OxiSelect^TM^ hydrogen peroxide were obtained from Immunodiagnostic Systems Inc. (Fountain Hills, AZ, USA), Biomedical Technologies Inc. (Stoughton, MA, USA), BioAssay Systems (Hayward, CA, USA), and Cell Biolabs Inc. (San Diego, CA, USA), respectively. Doxorubicin, 3-methladenine, MTT (3-(4,5-dimethylthiazol-2-yl)-2,5-diphenyltetrazolium bromide), a leukocyte acid phosphatase (TRAP) assay kit, toluidine blue, *N*-acetyl-l-cysteine (NAC), and diphenyliodonium (DPI) were purchased from Sigma Chemical (St. Louis, MO, USA). *N*-(Biotinoyl)-*N*′-(iodoacetyl) ethylenediamine (BIAM), and Mito-SOX red were purchased from Invitrogen (Carlsbad, CA, USA). Lipofectamine 3000 was obtained from Molecular Probes (Carlsbad, CA, USA). NE-PER nuclear and cytoplasmic extraction reagents were acquired from Pierce (Waltham, MA, USA). In this research, the following primary antibodies were used: p62 (H00008878-M01) from Abnova (Taipei, Taiwan), LC3B (#2775) and lamin B1 (#13435) from Cell Signaling (Danvers, MA, USA), transcription factor EB (TFEB; A303-673A) from Bethyl Laboratories Inc. (Montgomery, TX, USA), and β-actin (A5441) from Sigma Chemical. Thermo Fisher Scientific (Waltham, MA, USA) provided the 2′,7′-dichlorofluorescein diacetate (H_2_DCFDA), HRP-conjugated secondary Abs, and SYBR Green Real-Time PCR Master Mixes. Horseradish peroxidase (HRP)-streptavidin, small interfering RNA (siRNA) against ATG7 anTRPML1 (accession number: NM 053177), as well as scrambled siRNA (scRNA), were purchased from Santa Cruz Biotechnology (Dallas, TX, USA). M-MLV reverse transcriptase and SYBR Green Real-Time PCR Master Mixes were purchased from Promega (Madison, WI, USA). QIAzol reagent was acquired from Qiagen (Hilden, Germany). 

### 2.3. Animals and Study Design

C57BL/6J female mice, ten weeks old, obtained from The Jackson Laboratory (Hana, Busan, Korea) were randomly divided into the following four groups: vehicle control (*n* = 5), vehicle + 3-methyladenine (3-MA) (*n* = 5), doxorubicin (DOX, *n* = 5), and DOX + 3-MA (*n* = 5). Mice were intraperitoneally injected with DOX (5 mg/kg) dissolved in distilled water once per week for three weeks. The 3-MA was dissolved in DMSO and diluted with PBS to gain a DMSO concentration of 2%. The mice were given daily intraperitoneal injections of 5 mg/kg of 3-MA in 200 μL PBS (or just DMSO as vehicle control) every day for four weeks and sacrificed in a CO_2_ chamber. At the end of the experiments, femurs were scanned by micro-computed tomography (μCT) imaging system (SkyScan 1072, SkyScan, Kontich, Belgium) to measure bone mineral density (BMD) and microarchitecture, as described previously [6]. To examine TRAP-positive OCs In vivo, mouse femora were excised, cleaned with a soft paper tissue, and decalcified in EDTA. Representative histologic sections of the distal femoral metaphysis of mice injected with DOX with/without 3-MA were stained for TRAP to identify Ocs (original magnification × 200). Next, an in vivo marker of bone resorption, serum C-telopeptide fragments of collagen type 1 (CTX-1), was analyzed with the RatLaps EIA, according to the manufacturer’s instructions. Serum osteocalcin was measured with an osteocalcin EIA kit and serum alkaline phosphatase (ALP) was measured with a colorimetric kinetic assay. Serum hydrogen peroxide was evaluated using a colorimetric H_2_O_2_ assay kit, and serum MCP-1 was detected using a sandwich ELISA with the appropriate antibodies.

### 2.4. Cultures of Osteoblast and OC, and OC Formation

Primary osteoblasts were prepared from calvarias of C57BL/6J newborn mice, cultured in α-MEM containing 10% FBS for 3 d to confluence as described [22], and further incubated with DOX in osteogenic medium (10% FBS, 50 μg/mL ascorbic acid, and 10 mm β-glycopyrophosphate) to induce osteoblast differentiation. According to a prior report [23], bone marrow cells were extracted from C57BL/6J male mice. The femora and tibiae were aseptically removed and dissected without adhering soft tissue. Following the dissection of the bone, the marrow cavity was rinsed with α-MEM from one end of the bone using a sterile 21-gauge needle, and a single-cell suspension was prepared using a Pasteur pipette. M-CSF (30 ng/mL) was added to the cells for 16 h, after which floating cells were cultured for further 2 days. Nonadherent cells were washed and adherent bone marrow-derived macrophages (BMMs) were treated with M-CSF (30 ng/mL) and RANKL (40 ng/mL) for the times indicated in each figure. Then cells were fixed in 10% formalin for 10 min, and, as previously mentioned [23], cells were stained for tartrate-resistant alkaline phosphatase (TRAP). After counting all the multinucleated (three or more nuclei) TRAP-positive cells, more than 100 cells were chosen randomly to measure the area, maximum diameter, and fusion index [24].

### 2.5. Bone Resorption

To evaluate OC resorption capacity, cells were seeded on dentine slices as described previously [6]. To do this, mature Ocs were developed from BMMs with M-CSF (30 ng/mL) and RANKL (40 ng/mL) for 4 days. These cells were seeded on dentine slices and cultured for a further 3 days with RANKL and M-CSF in the presence or absence of DOX (2.5 nM). After being formalin-fixed, the cells were stained for TRAP. Afterward, to visualize resorption pits, the cells were removed by 1 M NH_4_OH and the dentine slices were dyed with 1% (*w*/*v*) toluidine blue in 0.5% sodium borate. The resorption pit area and TRAP-positive cells were analyzed by ImageJ (version 1.37) software (National Institutes of Health, Bethesda, MD, USA).

### 2.6. RNA Isolation and qPCR 

Total RNA was reverse-transcribed with random primers and M-MLV reverse transcriptase (Promega, Madison, WI, USA). Moreover, qPCR was conducted using SYBR Green 1 Taq polymerase (Qiagen, Hilden, Germany) and the appropriate primers on a StepOnePlus™ Real Time System (Applied Biosystems, Foster City, CA, USA). Primer specificity was confirmed by melting curve analysis and agarose gel electrophoresis. The housekeeping gene 18S rRNA (RPS; accession number: NM_012052) was amplified in parallel with genes of interest. Relative copy numbers compared to RPS were calculated using the 2^−ΔΔCt^ method. The experiments were conducted using the following primers: 5′-GAC CAC CTT GGC AAT GTC TCT G-3′ and 5′-TGG CTG AGG AAG TCA TCT GAG TTG-3′ (TRAP; accession number: NM_001102405); 5′-AAT AAC ATG CGA GCC ATC ATC-3′ and 5′-TCA CCC TGG TGT TCT TCC TC-3′ (NFAT2; accession number: NM_198429); 5′-AGT TGC CCT CTT ATG AAG GAG AAG-3′ and 5′-GGA GTG TCG TCC CAG CAC AT-3′ (calcitonin receptor; accession number: NM_001377018); 5′-GGG CCA GGA TGA AAG TTG TA-3′ and 5′-CAC TGC TCT CTT CAG GGC TT-3′ (Cathepsin K; accession number: NM_007802); 5′-TCC TCC ATG AAC AAA CAG TTC CAA-3′ and 5′-AGA CGT GGT TTA GGA ATG CAG CTC-3′ (DC-STAMP; accession number: NM_029422); 5′-TTC AGT TGC TAT CCA GGA CTC GGA-3′ and 5′-GCA TGT CAT GTA GGT GAG AAA TGT GCT CA-3′ (ATP6v0d2; accession number: NM_175406); 5′-AAG GCT TCT TCT TGC TGG TG-3′ and 5′-GCC TTA CCC TCA TGA TGT CC-3′ (ALP; accession number: NM_007431.3); 5′-AGA CTC CGG CGC TAC CTT-3′ and 5′-CTC GTC ACA AGC AGG GTT AAG-3′ (Osteocalcin; accession number: NM_007541.3); 5′-GTG CTC CTG GTA TTG CTG GT-3′ and 5′-GGC TCC TCG TTT TCC TTCTT-3′ (Col1a1; accession number: NM_007742.4); 5′-TTTAAACAAACACGATGATGATG-3′ and 5′-ATG GAC TTC CCC CTT CTTG-3′ (Osterix; accession number: NM_130458.4); 5′-CCA CCA CTC ACT ACC ACA CG-3′ and 5′-TCA GCG TCA ACA CCA TCA TT-3′ (RUNX2; accession number: NM_001146038.2). 

### 2.7. Apoptosis Assay

OC apoptosis was detected by annexin V staining as described previously [25]. BMMs were stimulated with M-CSF and RANKL for 72–80 h to differentiate mature Ocs. The mature Ocs were then washed and incubated with M-CSF and RANKL with or without DOX for 6 h. Next, the Ocs were harvested by incubation with 0.02% EDTA for 20 min, followed by calcium/magnesium-free PBS to scrape the cells. Finally, the cells were incubated with annexin V-FITC in annexin binding buffer for 15 min and immediately analyzed by FACSCalibur.

### 2.8. Fractionation and Western Blot Analysis

To examine the localization of TFEB, total proteins from cultured cells were extracted on ice with lysis buffer (50 mM Tris-HCl, pH 8.0, 150 mM NaCl, 1 mM EDTA, 0.5 percent Nonidet P-40, and 0.01 percent protease inhibitor mixture).

SDS-PAGE was used to separate the proteins and transferred to nitrocellulose membranes. After that, the membranes were blocked for 1 h at room temperature using 5% skim milk in Tris-buffered saline that contained 0.1% Tween-20. The following primary antibodies were incubated overnight at 4 °C: LC3, TFEB, lamin B1 (1:1000 each), and p62, β-actin (1:10,000 each), followed by reactions with HRP-conjugated secondary antibodies for 1 h, and then developed using chemiluminescent substrates.

### 2.9. Flow Cytometric Quantification of Acidic Vesicular Organelles with Acridine Orange Staining

Referring to a previous report [26], autolysosomes were stained with acridine orange (AO) (1 μg/mL) for 20 min to detect autophagy and analyzed by flow cytometry. AO is a cell-permeant nucleic acid binding dye that accumulates in acidic environments. In AO-stained cells, the cytoplasm and nucleolus appear bright green and dim red, respectively, whereas AVOs appear bright red. The intensity of red fluorescence is proportional to the acidity and volume of the AVO. 

### 2.10. Determination of Mitochondrial Reactive Oxygen Species (ROS) 

Intracellular ROS and mitochondria-generated ROS were detected using the fluorescent probe 2′,7′-dichlorofluorescein diacetate (H_2_DCFDA) and the Mito-SOX red mitochondrial superoxide indicator, respectively. BMMs were treated with M-CSF and RANKL with DOX for the time points indicated in the figure and then loaded with H_2_DCFDA or Mito-SOX red for 30 min at 37 °C. Using a FACS Calibur flow cytometer, intracellular and mitochondrial ROS production was assessed (Becton Dickinson, Franklin Lakes, NJ, USA).

### 2.11. Detection of Oxidized TRPML1 via Carboxymethylation

BMMs were incubated with M-CSF (30 mg/mL) and RANKL (40 mg/mL) in the presence of DOX for 72 h. The cells were lysed with 100 µM BIAM-containing lysis buffer (50 mM Tris-HCl, pH 7.5, 150 mM NaCl, 0.5% Triton X-100, 10 µg/mL aprotinin, and 10 µg/mL leupeptin; the buffer was rendered free of oxygen by bubbling nitrogen gas at a low flow rate for 20 min). The reduced form of cysteine was detected using a sulfhydryl modifying chemical, BIAM [27]. The lysate was separated by centrifugation and then processed to immunoprecipitation with 1 μg of TRPML1-specific antibodies. Immunocomplexes labeled with BIAM were identified using HRP-conjugated streptavidin and produced using an improved chemiluminescence kit.

### 2.12. Transfection of siRNA

Small interfering RNA (siRNA) against ATG7, TRPML1, or scrambled siRNA (scRNA) was transfected using lipofectamine 3000 into BMMs that had been treated with M-CSF and RANKL for 40 h. In summary, 50 μL of Opti-MEM culture medium was mixed with 3.75 μL of the lipofectamine 3000 reagent, 2.5 μL of siRNA (50 μM), or an equivalent amount of scRNA. After 15 min of incubation, the mixtures were treated to 2 × 10^5^ cells that had been already seeded in 24-well plates, and the cells were further incubated with M-CSF and RANKL in either the presence or absence of DOX for the times indicated in the figure legends.

### 2.13. Statistical Analyses

All values were reported as means ± standard deviation (SD). Student’s *t*-test was used to evaluate differences between samples and their corresponding controls. Differences between groups were assessed via one-way ANOVA followed by Bonferroni post-hoc tests. A *p*-value less than 0.05 was considered statistically significant. 

## 3. Results

### 3.1. DOX Enhances the Activity of Ocs

**Figure 1:**
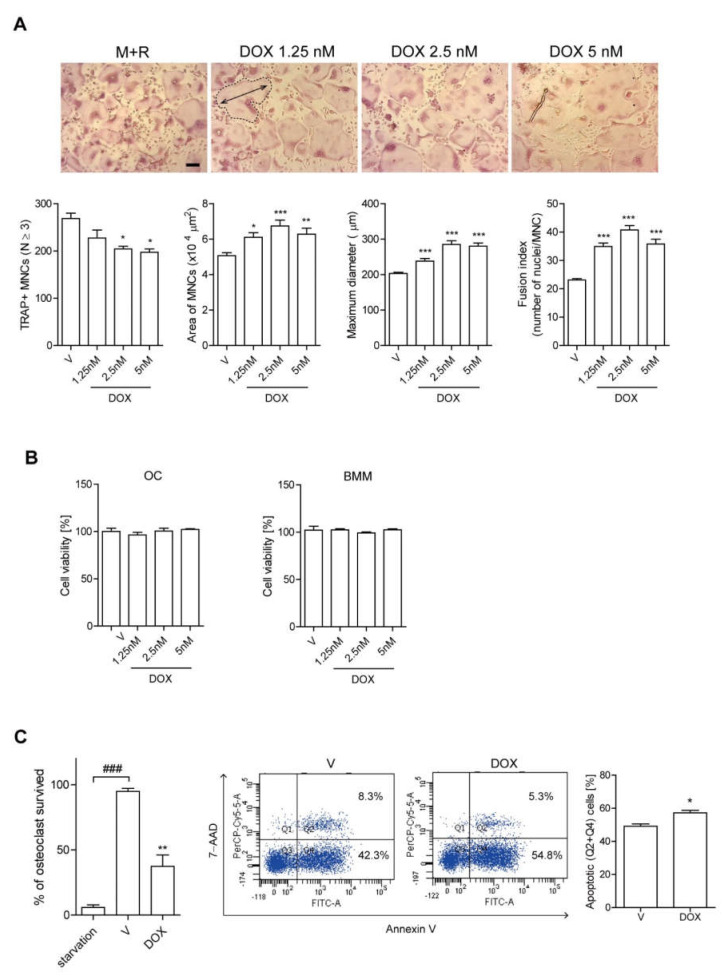

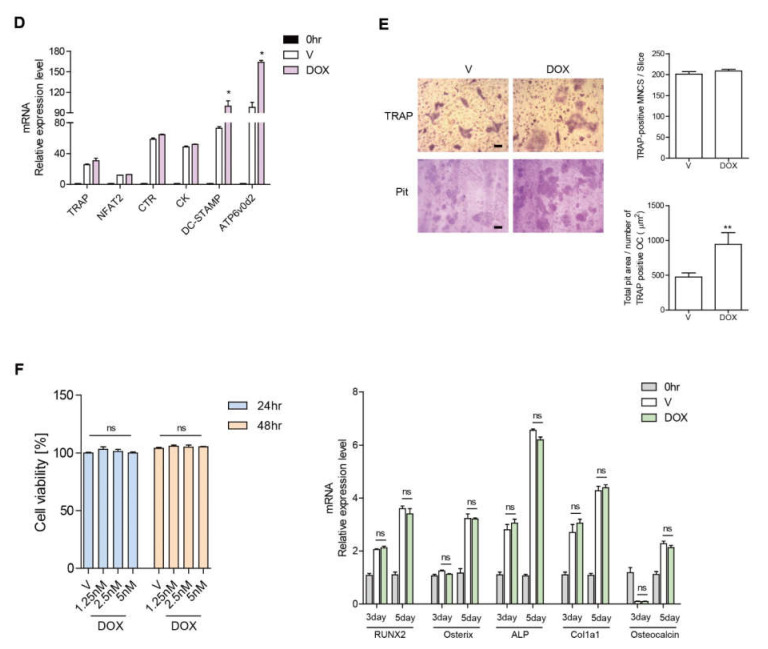
DOX enhances the activity of Ocs. (**A**,**B**) M-CSF and RANKL were treated in BMMs either with or without DOX (1.25, 2.5, 5 nM). After 72 h, the cells were fixed and more than 100 TRAP-positive MNCs from each culture were randomly selected. The produced Ocs’ area (bold line) and maximum diameter (double arrow) were measured. Fusion index was determined by counting the average number of nuclei per OC. Representative photos are shown (scale bar: 100 μm) (**A**). Cell viability was measured via the MTT assay in OC (left panel) and BMM (right panel) (**B**). (**C**) Mature Ocs were washed and incubated with M-CSF and RANKL with or without DOX for 6 h to measure TRAP-positive MNCs and annexin V-positive cells. (**D**) Using RNA extracted from cells treated with RANKL ± DOX (2.5 nM) for 48 h in the presence of M-CSF, OC-specific genes were analyzed by qPCR. The expression level before RANKL treatment was set to 1. (**E**) Mature OCs were cultured on dentine slices with M-CSF and RANKL ± DOX (2.5 nM) for 4 d, and cells were stained with TRAP to count number. Cells were then removed and dyed with toluidine blue. Representative photos of TRAP-positive OCs and resorption pits are shown (scale bar: 100 μm). The total pit area/number of TRAP-positive OCs was calculated. (**F**) primary calvarial osteoblasts were treated with DOX for 24 and 48 h for cell viability via MTT assay (left panel) and induced to differentiate with DOX in osteogenic medium for 3 and 5 d for the analysis of the indicated gene expression via qPCR (right panel). * *p* < 0.05; ** *p* < 0.01; *** *p* < 0.001 compared with RANKL alone-treated cells. ### *p* < 0.001 compared with the corresponding control. ns means no significance between them. Similar results were obtained in three independent sets of experiments.

To investigate the action mechanism of DOX in bone loss, we evaluated the effects of DOX on OC formation by counting TRAP-positive MNCs in vitro, since Ocs play a critical role in bone loss. DOX significantly increased OC surface area, maximum diameter, and the fusion index up to 2.5 nM, whereas the total number of TRAP-positive cells was decreased (Figure 1A). DOX did not induce any cytotoxicity in BMMs as well as Ocs under the assay conditions (Figure 1B). This discrepancy between increased OC area and decreased OC number induced by DOX prompted us to examine whether DOX affected OC survival. As expected, upon evaluating the portion of annexin V-positive cells (Figure 1C), our findings indicated that DOX reduced the fraction of OC that survived and enhanced OC apoptosis. Furthermore, no significant change was observed in the expression level of OC-specific genes, including TRAP, NFAT2, calcitonin receptor, and cathepsin K, whereas the expressions of DC-STAMP and ATP6v0d2 were increased in DOX-treated cells (Figure 1D). Mature Ocs stimulated with DOX in the presence of RANKL showed significantly higher total pit area/number of Ocs compared to cells stimulated with RANKL only (Figure 1E), demonstrating that DOX increased OC activity.

To investigate the effect of DOX on osteoblast, primary calvarial osteoblasts were treated with DOX before and after induction to differentiation in an osteogenic medium. No significant changes were observed by DOX in cell viability during proliferation and the expression of osteoblast-associated genes, Runx2, osterix, ALP, Col1a1, and osteocalcin during differentiation in osteoblasts (Figure 1F).

### 3.2. DOX Enhances Autophagy in OCs

**Figure 2:**
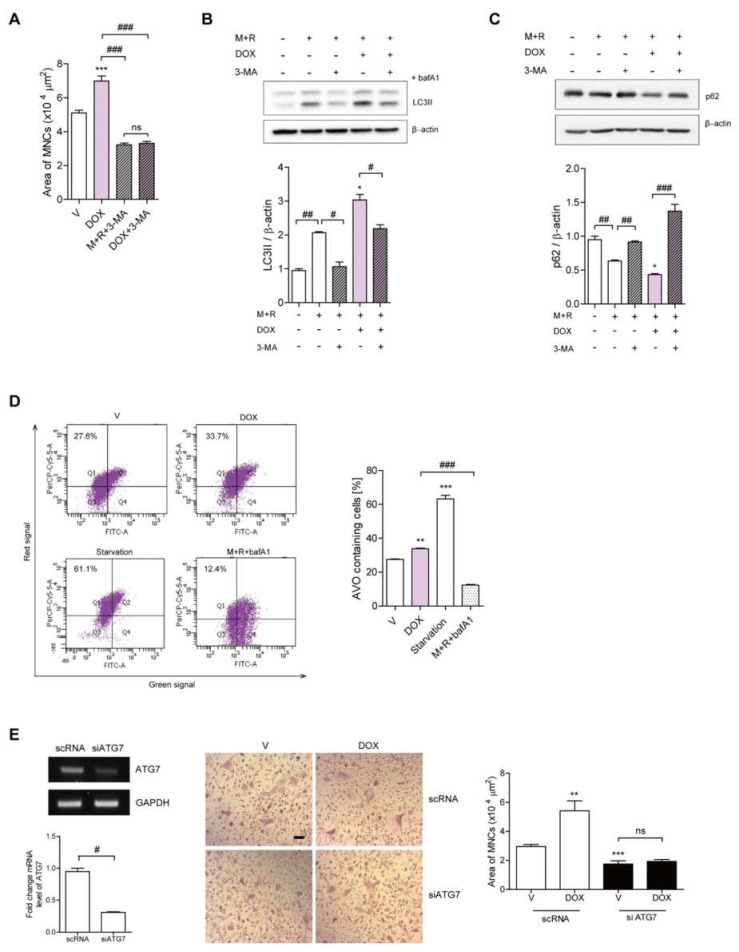
DOX enhances autophagy in OCs. (**A**–**D**) BMMs were incubated in the presence or absence of DOX (2.5 nM) ± 3-MA (0.5 mM) for 72 h. After TRAP staining, the area of OCs was measured (**A**). To block autophagic flux, the cells were treated with bafilomycin A1 (25 nM) for 4 h before harvesting and the LC3II levels were analyzed (**B**). The levels of p62 were analyzed (**C**). LC3II and p62 expression levels were normalized to ß-actin levels. (**D**) DOX-induced AVO formation was analyzed by flow cytometry. As positive controls, bafilomycin A1 was added and for negative controls, cells were incubated without FBS for 4 h for starvation. The values represent the percentage of AVO-containing cells. (**E**) BMMs were transfected with scRNA or siATG7 in the presence of DOX (2.5 nM) for 72 h. The downregulation of ATG7 by siRNA was confirmed by reverse transcription (RT)-PCR and qPCR. More than 100 TRAP-positive MNCs in each culture were randomly selected and OC area of each was analyzed. Representative photos are shown (scale bar: 100 μm). * *p* < 0.05; *** *p* < 0.001 compared with cells treated with RANKL alone. ** *p* < 0.01 compared with vehicle. # *p* < 0.05; ## *p* < 0.01; ### *p* < 0.001 compared with each corresponding control. ns means no significance between them. + and − mean “with and without”. Similar results were obtained in three independent sets of experiments.

Given that our previous data demonstrated that autophagy is closely associated with increasing OC area [7,8] and DOX has been reported to modulate autophagy in various cancer cells [20,28], we hypothesized that DOX may induce autophagy, thus affecting the activity of OCs. To assess whether the effects of DOX on OCs were due to autophagy induction, OCs were exposed to 3-MA, an autophagosome inhibitor. The addition of 3-MA significantly decreased the OC area that had been increased upon DOX stimulation, thus counteracting the effect of DOX (Figure 2A). Next, we evaluated whether DOX augmented autophagosome formation by immunoblotting cell lysates with an antibody against microtubule-associated protein light chain 3 (LC3). RANKL induced the lipidated form of LC3 (LC3II), a marker of autophagic flux, when coupled with bafilomycin A1, an antibiotic that blocks the fusion of the autophagosome with lysosomes and leads to the accumulation of LC3II (Figure 2A). DOX treatment markedly up-regulated LC3II, whereas 3-MA reversed this DOX-induced effect (Figure 2B). The level of p62/SQSTM1, which can be used as a surrogate marker of autophagy [29], was significantly reduced with DOX treatment, and this effect was reversed by 3-MA (Figure 2C). To assess whether DOX affected the autolysosome formation that was subsequently accompanied by autophagosome formation, the fraction of acidic vesicular organelles (AVOs; i.e., including autolysosome-containing cells) was determined by flow cytometry using the pH-sensitive acridine orange, fluorescent dye. As illustrated in Figure 2D, DOX significantly increased the portion of AVO-containing cells at 72 h. Starvation and bafilomycin A1 were used as positive and negative controls, respectively. The silencing of ATG7 reduced the OC area that had been increased upon RANKL (Figure 2E). Furthermore, the effect of DOX on OC area was abolished when ATG7 was knocked down (Figure 2E).

### 3.3. DOX Increases ROS Levels, Leading to Increased TFEB Nuclear Translocation in OCs

**Figure 3:**
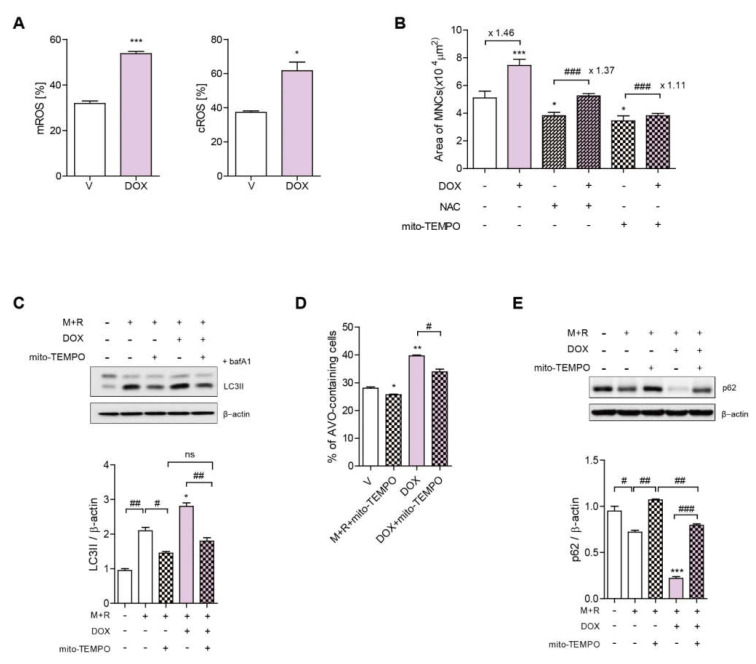
DOX increases mROS levels to induce autophagy, resulting in increased OC area. BMMs were incubated with M-CSF and RANKL with the indicated conditions (DOX, 2.5 nM; Mito-TEMPO, 300 nM; NAC 3 mM). (**A**) Mitochondrial ROS was determined by flow cytometry using Mito-SOX after 24 h of incubation, and cytosolic ROS was determined after 48 h of exposure using H_2_DCF-DA. (**B**) After 72 h, the cells were fixed to evaluate the OC area of more than 100 randomly selected TRAP-positive MNCs. (**C**,**E**) The cell lysates were subjected to western blot analysis to determine the expression levels of LC3II with the addition of bafilomycin A1 (25 nM) for 4 h and p62. The quantified levels of LC3II and p62 were shown normalized to β-actin. (**D**) Cells with staining for AVOs were monitored using flow cytometry. * *p* < 0.05; ** *p* < 0.01; *** *p* < 0.001 compared with cells treated with RANKL alone. # *p* < 0.05; ## *p* < 0.01; ### *p* < 0.001 compared with each corresponding control. The number above the line between histograms is a ratio for each group. Similar results were obtained in three independent sets of experiments. ns means no significance between them. + and − mean “with and without”.

Our previous studies, as well as those of other groups, have identified mitochondrial ROS (mROS) as a critical trigger for autophagy in OCs [7,30]. Excessively high ROS levels have been reported to be a major mechanism of action of DOX, leading to mitochondrial dysfunction and damage to the vascular endothelium [31]. Based on these observations, we hypothesized that DOX may increase mROS to up-regulate autophagy in OCs. As expected, DOX markedly increased mROS and cytosolic ROS (cROS) after 24 h and 48 h, respectively (Figure 3A). Next, to investigate the contribution of ROS to the effect of DOx on OCs, DOX-stimulated OCs were treated with Mito-TEMPO, an mROS scavenger, and *N*-acetyl-l-cysteine (NAC), a ROS scavenger. Mito-TEMPO abolished the increase in the OC area induced by DOX, whereas NAC attenuated it (Figure 3B). The level of LC3II and the fraction of AVO-containing cells that were increased upon DOX stimulation were also dramatically reduced by Mito-TEMPO (Figure 3C,D). Moreover, Mito-TEMPO attenuated the level of p62 that was decreased by DOX (Figure 3E).

### 3.4. Dox Induces Oxidation of TRPML1 and Nuclear Localization of TFEB

**Figure 4:**
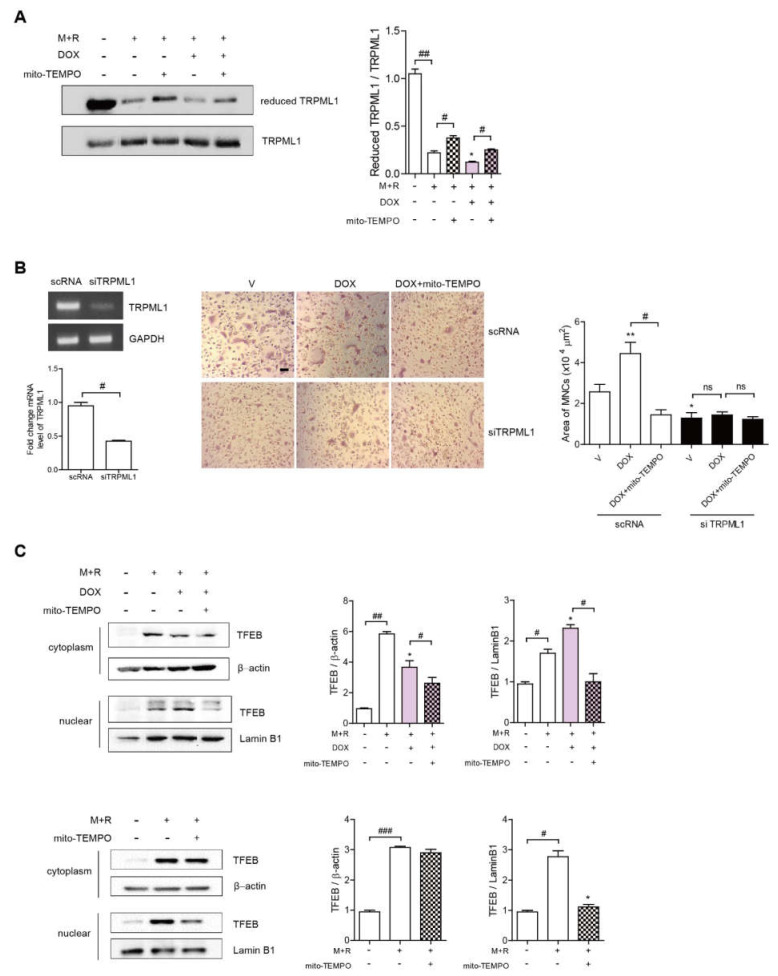
DOX increases TFEB nuclear translocalization via TRPML1 by upregulating mROS levels in OCs. (**A**,**C**) BMMs were incubated in the presence or absence of DOX (2.5 nM) ± Mito-TEMPO (300 nM) with M-CSF and RANKL. (**A**) After 72 h, the cell lysates were labeled with *N*-(biotinoyl)-*N*′-(iodoacetyl) ethylenediamine, and TRPML1 was immunoprecipitated (IP) from each sample. To assess the reduced form of TRPML1, HRP-streptavidin immunoblotting was used. (**B**) The cells were transfected with 50 nM of scRNA or siTRPML1 and incubated further for 72 h upon stimulation with DOX ± Mito-TEMPO (300 nM) in the presence of M-CSF and RANKL. The siRNA-mediated silencing of TRPML1 was confirmed by RT-PCR and qPCR. After fixation, more than 100 TRAP-positive MNCs in each culture were randomly selected to determine the area of the formed OCs. Scale bar: 100 μm. (**C**) Western blot analysis of cytoplasmic and nuclear fractions from cultured cells using anti-TFEB antibodies. Expression levels were normalized to β-actin (cytoplasmic extract) or lamin B1 (nuclear fraction). * *p* < 0.05; ** *p* < 0.01 compared with cells treated with RANKL only. # *p* < 0.05; ## *p* < 0.01; ### *p* < 0.001 compared with each corresponding control. ns means no significance between them. + and − mean “with and without”. Similar results were obtained in three independent sets of experiments.

The oxidation of TRPML1 (a member of the transient receptor potential cation channel family and mucolipin subfamily) by mROS has been reported to facilitate the nuclear localization of transcription factor EB (TFEB) [7,30]. Therefore, we next sought to determine whether the oxidation of TRPML1 was mediated by DOX stimulation. As shown in Figure 4A, RANKL only decreased the level of reduced TRPML1, whereas Mito-TEMPO reversed this effect. Moreover, the reduced TRPML1 was lowered further by DOX, whereas Mito-TEMPO recovered it. Next, TRPML1 was silenced to confirm that the effect of DOx on OCs was mediated by changes in the expression of this gene. Down-regulation of TRPML1 markedly reduced the OC area that was increased upon stimulation by RANKL alone or combined with DOX (Figure 4B). The enhanced OC area by DOX was abolished and no further decrease was found with the addition of Mito-TEMPO when TRPML1 was knocked down (Figure 4B). Next, we investigated whether DOX facilitated TFEB nuclear translocation stimulated by RANKL alone. DOX stimulation fortified the translocation of TFEB in the nucleus, whereas the addition of Mito-TEMPO markedly attenuated it (Figure 4C). Mito-TEMPO also reduced nuclear localization of TFEB upon exposure to RANKL alone (Figure 4C).

### 3.5. Doxorubicin Induces Bone Loss Accompanied by Increased Oxidative Stress

**Figure 5:**
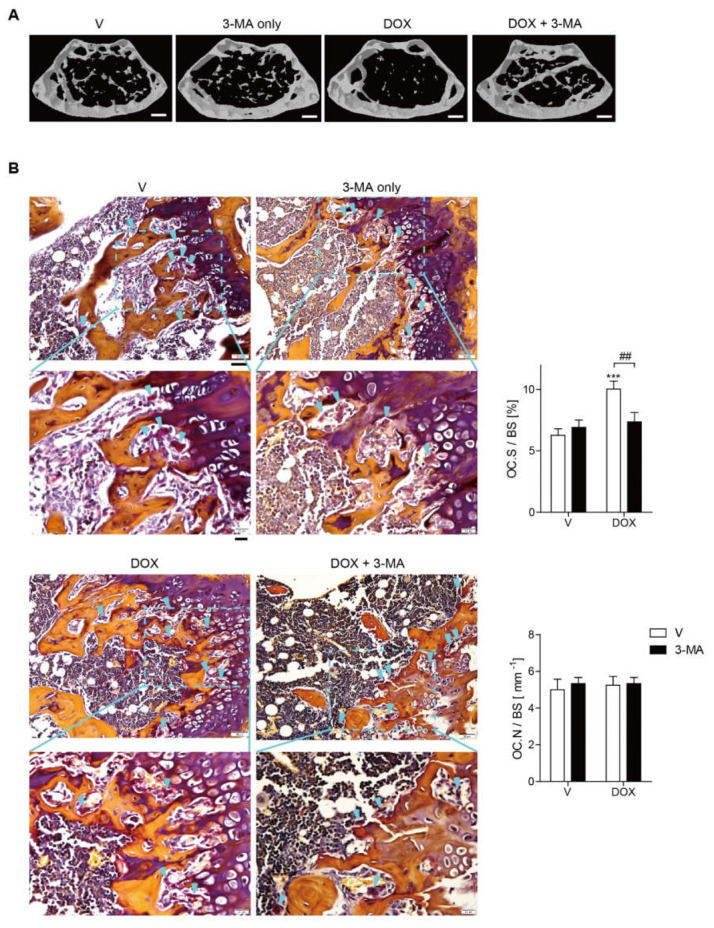
Doxorubicin induces bone loss accompanied by increased oxidative stress. (**A**) Representative μCT images of distal femora 1.0 mm from the growth plate of mice treated with vehicle (*n* = 5), DOX (5 mg/kg/week) (*n* = 5), DOX + 3-MA (5 mg/kg/d) (*n* = 5), or 3-MA only (5 mg/kg/d) (*n* = 5). Mice were treated for four weeks starting at the age of 10 weeks. Scale bar: 2 mm. (**B**) To examine TRAP-positive OCs in vivo, mouse femora were decalcified in EDTA. Representative histological sections of the distal femoral metaphysis of mice from each of the four groups were stained for TRAP to identify OCs (indicated by arrowheads). The acquired data were then used to obtain the OC.S/BS ratio (OC surface area divided by total bone surface area) and the OC.N/BS ratio (OC number divided by total bone surface). The images in the bottom panel are partially enlarged images of the squared fields of the pictures. Upper panels; scale bar = 50 µm. Lower panels; scale bar = 20 µm. *** *p* < 0.001 compared with vehicle-treated mice. ## *p* < 0.01 compared with DOX-treated mice. Differences between groups were analyzed by two-way ANOVA, followed by Bonferroni post-hoc tests to compare the effect of DOX (OC.S/BS; *p* < 0.01, OC.N/BS; not significant), the effect of 3-MA (OC.S/BS and OC.N/BS; not significant), and their interaction (OC.S/BS; *p* < 0.05, OC.N/BS; not significant). Similar results were obtained in three independent sets of experiments. The μCT analysis was performed with the representative set.

Our in vitro data suggested that DOX augmented the activity of OCs through enhancing autophagy, we assessed the in vivo effect of DOX in mice with 3-MA, an autophagy inhibitor. To investigate the mechanism of doxorubicin (DOX)-induced bone loss, µCT scans of femurs from mice injected 3-MA or vehicle after treatment with DOX were analyzed. The administration of DOX at a 5 mg/kg dose for 3 weeks resulted in bone loss, whereas combined treatment of 3-MA at a dose of 5 mg/kg for 4 weeks had a significant protective effect against DOX-induced bone loss (Figure 5A). No significant changes in body weight were observed among the four groups at 14 weeks of age (data not shown). Our µCT analysis indicated that DOX induced significant bone loss with decreased bone mineral density (BMD), bone volume (BV/TV), and trabecular thickness (Tb. Th), as well as increased trabecular spaces (Tb. Sp.), compared to the vehicle control (Figure 5A and Table 1). In contrast, the injection of 3-MA reversed the above-described effects on bone parameters induced by DOX (Figure 5A, Table 1), whereas 3-MA alone did not elicit any significant differences compared with the vehicle control (Figure 5A, Table 1). In vivo TRAP staining showed that the femur from DOX-treated mice increased the OC surface area indicated as OC.S./BS ratio (the ratio of OC surface to total bone surface area), compared to that from vehicle-treated mice. Enlarged OC surface area had been significantly reduced by injection of 3-MA in DOX-injected mice (Figure 5B). However, neither DOX nor 3-MA affected the number of OCs denoted as OC.N./BS ratio (the ratio of OC number to total bone surface area) (Figure 5B). Consistently, an in vivo bone resorption marker CTX-1 was up-regulated upon DOX treatment alone. The 3-MA co-treatment markedly attenuated this effect, whereas no significant difference was found with the injection of 3-MA alone. Neither DOX nor 3-MA affected in vivo bone formation markers, serum ALP, or osteocalcin compared with the vehicle (Table 1). The same patterns were observed with the number of osteoblasts identified by H&E staining (Supplementary Figure S1). DOX alone increased the serum MCP-1 levels that had decreased upon 3-MA treatment (Table 1). Serum ROS levels were also measured to determine whether DOX exacerbated oxidative stress. DOX alone induced a significant increase in serum ROS, whereas the combined treatment of 3-MA dramatically attenuated this effect (Table 1).

## 4. Discussion

Clinical studies have reported a significant link between DOX used to treat cancer patients and bone loss [13,14]. The effect of DOX on bone loss has also been reported in animal models [18,19], but its action mechanisms have not been clearly elucidated. 

Our findings demonstrated that DOX markedly increases the activity of OCs, resulting in bone loss. Ovarian cancer patients are commonly treated with a DOX dose of 40–60 mg/m^2^ [32]. Therefore, the DOX in our in vivo study (5 mg/kg or 15 mg/m^2^) [33] is within a plausible range to induce various side effects associated with DOX in animal studies. The administration of 3-MA, an autophagy inhibitor, reversed DOX-induced bone loss but did not affect the vehicle-treated group in vivo, suggesting that autophagy plays a critical role in bone loss. In vivo TRAP staining demonstrated that the increase in OC surface/bone surface ratio induced by DOX was decreased by 3-MA without affecting the number of OCs, suggesting that the effect of DOX is mainly mediated by an increase in the activity of OCs. In vivo, neither DOX nor 3-MA changed bone formation based on serum bone formation markers, as well as the number of osteoblasts under our experimental conditions. Consistently, in vitro studies also showed that no significant changes of cell viability or the expression of osteoblast differentiation-associated genes were observed in primary calvarial osteoblasts with DOX. These results indicated that OCs played a major role in DOX-induced bone loss. In contrast, Yao et al. [18] reported that DOX enhanced bone formation in INK-ATTAC transgenic mice in which p16-expressing senescent cells were eliminated in an inducible manner. Additionally, the effect of autophagy on bone formation is also a controversial topic. In agreement with our results, inhibition of autophagy by 3-MA did not affect bone formation in ovariectomy-induced bone loss [34], whereas osteoblast-specific autophagy-deficient mice exhibited a significant reduction in trabecular bone mass [35]. 

The limitation of our studies was that the effect of DOX or 3-MA on in vivo bone formation has not been investigated due to unavailability of equipment necessary for it. To investigate the molecular mechanism of DOX activity in OCs, OCs were treated with DOX at a 2.5–5.0 nM range. Assuming that the plasma level is equivalent to the level in bone tissue, the injected DOX dose of 5 mg/kg could achieve a plasma level of <5 ng/mL [36], which is slightly lower than the concentrations in our in vitro studies. 

In agreement with the in vivo studies, DOX increased the OC area and OC activity in dentine slices, whereas it decreased OC number and did not affect the expression of genes associated with OC differentiation in vitro. Additionally, DOX induced apoptosis of OCs, thus decreasing overall OC survival rates. These results suggested that the effect of DOX is likely attributed to an increase in OC activity despite a reduction in OC numbers.

DOX also increased autophagosome formation, as demonstrated by increases in LC3II coupled with decreases in p62. DOX stimulation also increased fusion with lysosomes via an increase in the fraction of AVO-containing OCs. The 3-MA, which impairs autophagosome formation as a selective phosphoinositide 3-kinase inhibitor, and knock-down of ATG7 completely abolished the DOX-induced increase in OC area, which could be an indirect indication for OC activity. These findings suggested that the effect of DOX on OC activity was mainly due to accelerated autophagosome formation in OCs. 

DOX induced oxidative stress in vivo and increased mROS levels in OCs in vitro. Mito-TEMPO reduced the DOX-induced increases in OC area, indicating that mROS mediated the effect of DOX on OCs. Our previous studies demonstrated that mROS induced the oxidation of TRPML1 in lysosomes, leading to TFEB nuclear localization [7]. Therefore, we assessed whether DOX followed this signaling pathway in OCs. The silencing of TRPML1 completely abolished the effects of DOX on OC area, suggesting that TRPML1 is an important mediator of this effect. DOX enhanced nuclear localization of TFEB, as demonstrated by western blot analysis after fractionation. In contrast, the addition of Mito-TEMPO attenuated this effect, confirming the role of mROS in the nuclear localization of TFEB induced by DOX in OCs. The up-regulation of autophagy by DOX was due to an increased level of mROS in OCs that oxidized TRPML1 on the lysosomal membrane, leading to nuclear localization of TFEB. DOX-induced autophagy has also been reported to contribute to the development of cardiomyopathy [30] and drug resistance in cancer patients [29].

**Figure 6:**
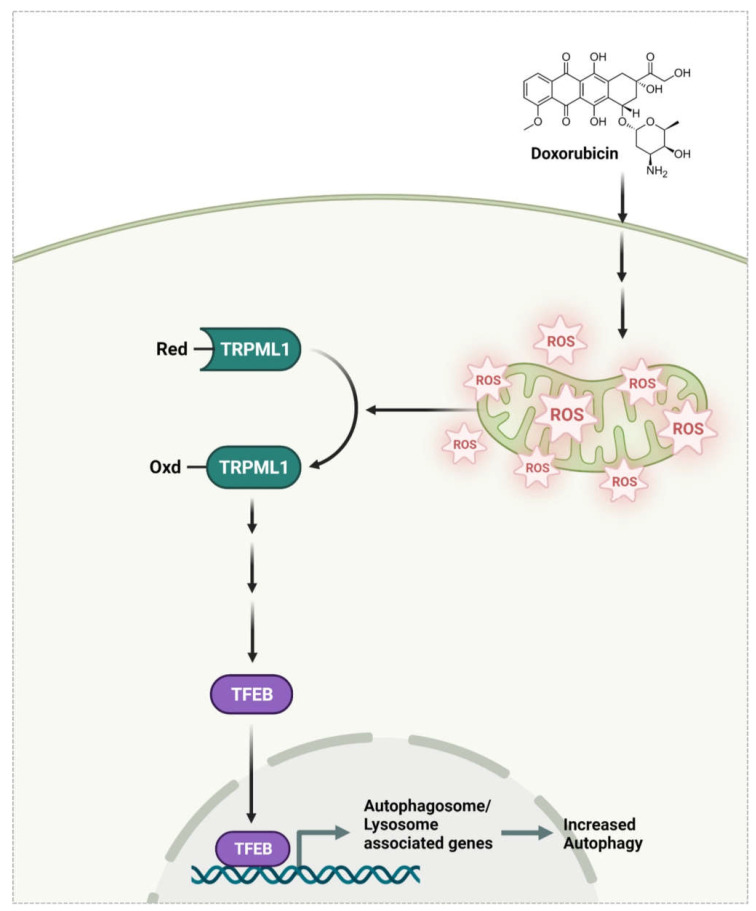
DOX induces autophagy via an axis of mROS/TRPML1/TFEB, resulting in enhanced activity of OCs. DOX increased mROS, which was responsible for the oxidation of TRPML1, leading to TFEB nuclear localization.

Taken together, our findings indicated that DOX-induced bone loss is at least partly due to an increase in autophagy in OCs via an axis of mROS/TRPML1/TFEB (Figure 6). Additionally, our findings suggest that modulation of autophagy could be a promising therapeutic strategy to reduce DOX-induced bone loss. Current treatment for bone loss due to chemotherapy involves bisphosphonates, estrogen, and denosumab [36]. However, several factors limit their use; estrogen cannot be used in hormone-related cancer patients in case of other side effects, bisphosphonate is associated with osteonecrosis of the jaw [37], and denosumab showed atypical femur fracture and increased bone turnover when treatment stopped [38]. Consequently, the inhibition of autophagy could show the alternative to protect from bone loss for patients to whom current treatment cannot be applied. 

## 5. Conclusions

Our studies have demonstrated that DOX-induced bone loss is caused by augmented bone resorption, at least partly due to increased autophagy in OCs via an axis of mROS/TRPML1/TFEB, suggesting that modulation of autophagy could be a promising therapeutic strategy to reduce DOX-induced bone loss. 

## Figures and Tables

**Table 1:** Trabecular microarchitecture and biochemical markers of DOX with and without 3-MA treatment in mice.

Parameter	DMSO		DOX	
	Vehicle	3-MA	Vehicle	3-MA
BMD [mg/cm^3^]	172.9 ± 3.21	176.2 ± 3.75	122.7 ± 3.24 ^a″^	170.5 ± 5.60 ^b″^
BV/TV [%]	15.57 ± 0.40	15.74 ± 0.29	11.25 ± 0.26 ^a″^	15.38 ± 0.47 ^b″^
Tb.Th [μm]	71.04 ± 1.88	71.15 ± 1.68	57.54 ± 1.05 ^a″^	65.37 ± 1.28 ^b^
Tb.Sp [μm]	384.7 ± 11.12	368.0 ± 14.09	672.9 ± 30.68 ^a″^	361.7 ± 19.43 ^b″^
ALP [U/L]	51.54 ± 0.66	51.64 ± 1.26	53.20 ± 2.19	53.40 ± 0.69
OCN [ng/mL]	25.29 ± 1.53	26.30 ± 1.03	27.11 ± 0.87	27.48 ± 0.76
CTX-1 [ng/mL]	25.34 ± 1.51	22.98 ± 0.89	45.23 ± 1.82 ^a″^	32.93 ± 1.49 ^b″^
MCP-1 [pg/mL]	139.2 ± 13.29	129.5 ± 10.84	294.1 ± 15.40 ^a″^	219.4 ± 12.66 ^b^
H_2_O_2_ [μM]	58.18 ± 0.80	54.81 ± 1.39	90.75 ± 2.12 ^a″^	76.44 ± 4.67 ^b^

DMSO (*n* = 5); 3-MA only (dissolved in DMSO, 5 mg/kg) (*n* = 5); DOX (dissolved in PBS, 5 mg/kg) (*n* = 5); DOX + 3-MA (*n* = 5). Data are represented as mean ± SD. Differences between groups were determined by two-way ANOVA, followed by Bonferroni post-hoc tests to compare the effects of 3-MA (Tb.Th and MCP-1; *p* < 0.05, CTX-1; *p* < 0.01, BMD, BV/TV and Tb.Sp; *p* < 0.001), DOX (Tb.Th; *p* < 0.05, MCP-1; *p* < 0.01, BMD, BV/TV, Tb.Sp and CTX-1; *p* < 0.001), and their interactions (BMD, BV/TV, Tb.Th, Tb.Sp, CTX-1, MCP-1 and H_2_O_2_; *p* < 0.001). ^a″^ *p* < 0.001 compared with DMSO-injected mice. ^b^ *p* < 0.05, ^b″^ *p* < 0.001 compared with DOX-injected mice.

## Data Availability

All original images and data are contained within the article and supplementary materials.

## Supplementary Materials

The following supporting information can be downloaded at: https://www.mdpi.com/article/10.3390/antiox11081476/s1, Figure S1: Representative images of hematoxylin and eosin (H&E) staining in distal femora from each of the four groups.

## References

[1] Parzych K.R., Klionsky D.J. (2014). An overview of autophagy: Morphology, mechanism, and regulation. Antioxid. Redox Signal..

[2] Zhang L., Guo Y.F., Liu Y.Z., Liu Y.J., Xiong D.H., Liu X.G., Wang L., Yang T.L., Lei S.F., Guo Y. (2010). Pathway-based genome-wide association analysis identified the importance of regulation-of-autophagy pathway for ultradistal radius BMD. J. Bone Miner. Res..

[3] Helfrich M.H., Hocking L.J. (2008). Genetics and aetiology of Pagetic disorders of bone. Arch. Biochem. Biophys..

[4] DeSelm C.J., Miller B.C., Zou W., Beatty W.L., van Meel E., Takahata Y., Klumperman J., Tooze S.A., Teitelbaum S.L., Virgin H.W. (2011). Autophagy proteins regulate the secretory component of osteoclastic bone resorption. Dev. Cell.

[5] Chung Y.H., Yoon S.Y., Choi B., Sohn D.H., Yoon K.H., Kim W.J., Kim D.H., Chang E.J. (2012). Microtubule-associated protein light chain 3 regulates Cdc42-dependent actin ring formation in osteoclast. Int. J. Biochem. Cell Biol..

[6] Park H.J., Son H.J., Sul O.J., Suh J.H., Choi H.S. (2018). 4-Phenyl butyric acid protects against lipopolysaccharide-induced bone loss by modulating autophagy in osteoclasts. Biochem. Pharmacol..

[7] Park H.J., Gholam-Zadeh M., Suh J.H., Choi H.S. (2019). Lycorine attenuates autophagy in osteoclasts via an axis of mROS/TRPML1/TFEB to reduce LPS-induced bone loss. Oxid. Med. Cell Longev..

[8] Sul O.J., Li G., Kim J.E., Kim E.S., Choi H.S. (2021). 7-ketocholesterol enhances autophagy via the ROS-TFEB signaling pathway in osteoclasts. J. Nutr. Biochem..

[9] Malla S., Niraula N.P., Singh B., Liou K.K., Sohng J.K. (2010). Limitations in doxorubicin production from *Streptomyces peucetius*. Microbiol. Res..

[10] Keizer H.G., Pinedo H.M., Schuurhuis G.J., Joenje H. (1990). Doxorubicin (adriamycin): A critical review of free radical-dependent mechanisms of cytotoxicity. Pharmacol. Ther..

[11] Bodley A., Liu L.F., Israel M., Seshadri R., Koseki Y., Giuliani F.C., Kirschenbaum S., Silber R., Potmesil M. (1989). DNA Topoisomerase II-mediated interaction of doxorubicin and daunorubicin congeners with DNA. Cancer Res..

[12] Chatterjee K., Zhang J., Honbo N., Karliner J.S. (2010). Doxorubicin cardiomyopathy. Cardiology.

[13] Hadji P., Ziller M., Maskow C., Albert U., Kalder M. (2009). The influence of chemotherapy on bone mineral density, quantitative ultrasonometry and bone turnover in pre-menopausal women with breast cancer. Eur. J. Cancer.

[14] Cameron D.A., Douglas S., Brown J.E., Anderson R.A. (2010). Bone mineral density loss during adjuvant chemotherapy in pre-menopausal women with early breast cancer: Is it dependent on oestrogen deficiency?. Breast Cancer Res. Treat..

[15] Shusterman S., Meadows A.T. (2000). Long term survivors of childhood leukemia. Curr. Opin. Hematol..

[16] Young D.M., Fioravanti J.L., Olson H.M., Prieur D.J. (1975). Chemical and morphologic alterations of rabbit bone induced by adriamycin. Calcif. Tissue Res..

[17] Fan C., Georgiou K.R., McKinnon R.A., Keefe D.M.K., Howe P.R.C., Xian C.J. (2016). Combination chemotherapy with cyclophosphamide, epirubicin and 5-fluorouracil causes trabecular bone loss, bone marrow cell depletion and marrow adiposity in female rats. J. Bone Miner. Metab..

[18] Yao Z., Murali B., Ren Q., Luo X., Faget D.V., Cole T., Ricci B., Thotala D., Monahan J., van Deursen J.M. (2020). Therapy-induced senescence drives bone loss. Cancer Res..

[19] Fonseca H., Carvalho A., Esteves J., Esteves V.I., Moreira-Gonçalves D., Duarte J.A. (2016). Effects of doxorubicin administration on bone strength and quality in sedentary and physically active Wistar rats. Osteoporos. Int..

[20] Chen C., Lu L., Yan S., Yi H., Yao H., Wu D., He G., Tao X., Deng X. (2018). Autophagy and doxorubicin resistance in cancer. Anticancer Drugs.

[21] Sciarretta S., Maejima Y., Zablocki D., Sadoshima J. (2018). The role of autophagy in the heart. Annu. Rev. Physiol..

[22] Sul O.J., Sung Y.B., Rajasekaran M., Ke K., Yu R., Back S.H., Choi H.S. (2018). MicroRNA-155 induces autophagy in osteoclasts by targeting transforming growth factor β-activated kinase 1-binding protein 2 upon lipopolysaccharide stimulation. Bone.

[23] Ke K., Safder M.A., Sul O.J., Kim W.K., Suh J.H., Joe Y., Chung H.T., Choi H.S. (2015). Hemeoxygenase-1 maintains bone mass via attenuating a redox imbalance in osteoclast. Mol. Cell Endocrinol..

[24] Okayasu M., Nakayachi M., Hayashida C., Ito J., Kaneda T., Masuhara M., Suda N., Sato T., Hakeda Y. (2012). Low-density lipoprotein receptor deficiency causes impaired osteoclastogenesis and increased bone mass in mice because of defect in osteoclastic cell-cell fusion. J. Biol. Chem..

[25] Sul O.J., Rajasekaran M., Park H.J., Suh J.H., Choi H.S. (2019). MicroRNA-29b enhances osteoclast survival by targeting Bcl2-modifying factor after lipopolysaccharide stimulation. Oxid. Med. Cell Longev..

[26] Bil J., Winiarska M., Nowis D., Bojarczuk K., Dabrowska-Iwanicka A., Basak G.W., Sułek K., Jakobisiak M., Golab J. (2010). Bortezomib modulates surface CD20 in B-cell malignancies and affects rituximab-mediated complement-dependent cytotoxicity. Blood.

[27] Giannoni E., Buricchi F., Raugei G., Ramponi G., Chiarugi P. (2005). Intracellular reactive oxygen species activate Src tyrosine kinase during cell adhesion and anchorage-dependent cell growth. Mol. Cell Biol..

[28] Tan Q., Wang H., Hu Y., Hu M., Li X., Aodengqimuge, Ma Y., Wei C., Song L. (2015). Src/STAT3-dependent heme oxygenase-1 induction mediates chemoresistance of breast cancer cells to doxorubicin by promoting autophagy. Cancer Sci..

[29] Sharifi M.N., Mowers E.E., Drake L.E., Macleod K.F. (2015). Measuring autophagy in stressed cells. Methods Mol. Biol..

[30] Zhang X., Cheng X., Yu L., Yang J., Calvo R., Patnaik S., Hu X., Gao Q., Yang M., Lawas M. (2016). MCOLN1 is a ROS sensor in lysosomes that regulates autophagy. Nat. Commun..

[31] He H., Wang L., Qiao Y., Zhou Q., Li H., Chen S., Yin D., Huang Q., He M. (2020). Doxorubicin induces endotheliotoxicity and mitochondrial dysfunction via ROS/eNOS/NO pathway. Front. Pharmacol..

[32] Gotlieb W.H., Bruchim I., Ben-Baruch G., Davidson B., Zeltser A., Andersen A., Olsen H. (2007). Doxorubicin levels in the serum and ascites of patients with ovarian cancer. Eur. J. Surg. Oncol..

[33] Saadh M.J., Haddad M., Dababneh M.F., Bayan M.F., Al-Jaidi B.A. (2020). A guide for estimating the maximum safe starting dose and conversion it between animals and humans. Syst. Rev. Pharm..

[34] Cheng L., Zhu Y., Ke D., Xie D. (2020). Oestrogen-activated autophagy has a negative effect on the anti-osteoclastogenic function of oestrogen. Cell. Prolif..

[35] Nollet M., Santucci-Darmanin S., Breuil V., Al-Sahlanee R., Cros C., Topi M., Momier D., Samson M., Pagnotta S., Cailleteau L. (2014). Autophagy in osteoblasts is involved in mineralization and bone homeostasis. Autophagy.

[36] Mosure K.W., Henderson A.J., Klunk L.J., Knipe J.O. (1997). Disposition of conjugate-bound and free doxorubicin in tumor-bearing mice following administration of a BR96-doxorubicin immunoconjugate (BMS 182248). Cancer Chemother. Pharmacol..

[37] Khosla S., Burr D., Cauley J., Dempster D.W., Ebeling P.R., Felsenberg D., Gagel R.F., Gilsanz V., Guise T., Koka S. (2007). Bisphosphonate-associated osteonecrosis of the jaw: Report of a task force of the American Society for Bone and Mineral Re-search. J. Bone Miner. Res..

[38] Lewiecki E.M. (2018). New and emerging concepts in the use of denosumab for the treatment of osteoporosis. Ther. Adv. Musculoskelet. Dis..

