# Supplementary material for: Doxorubicin Induces Bone Loss by Increasing Autophagy through a Mitochondrial ROS/TRPML1/TFEB Axis in Osteoclasts
Source: Antioxidants (Basel). 2022 Jul 28;11(8):1476. doi: 10.3390/antiox11081476 (PMC9404930; doi:10.3390/antiox11081476)
Supplement: Supplementary file 1 [file antioxidants-11-01476-s001.zip › antioxidants-1793655-supplementary.pdf]

antioxidants

MDPI

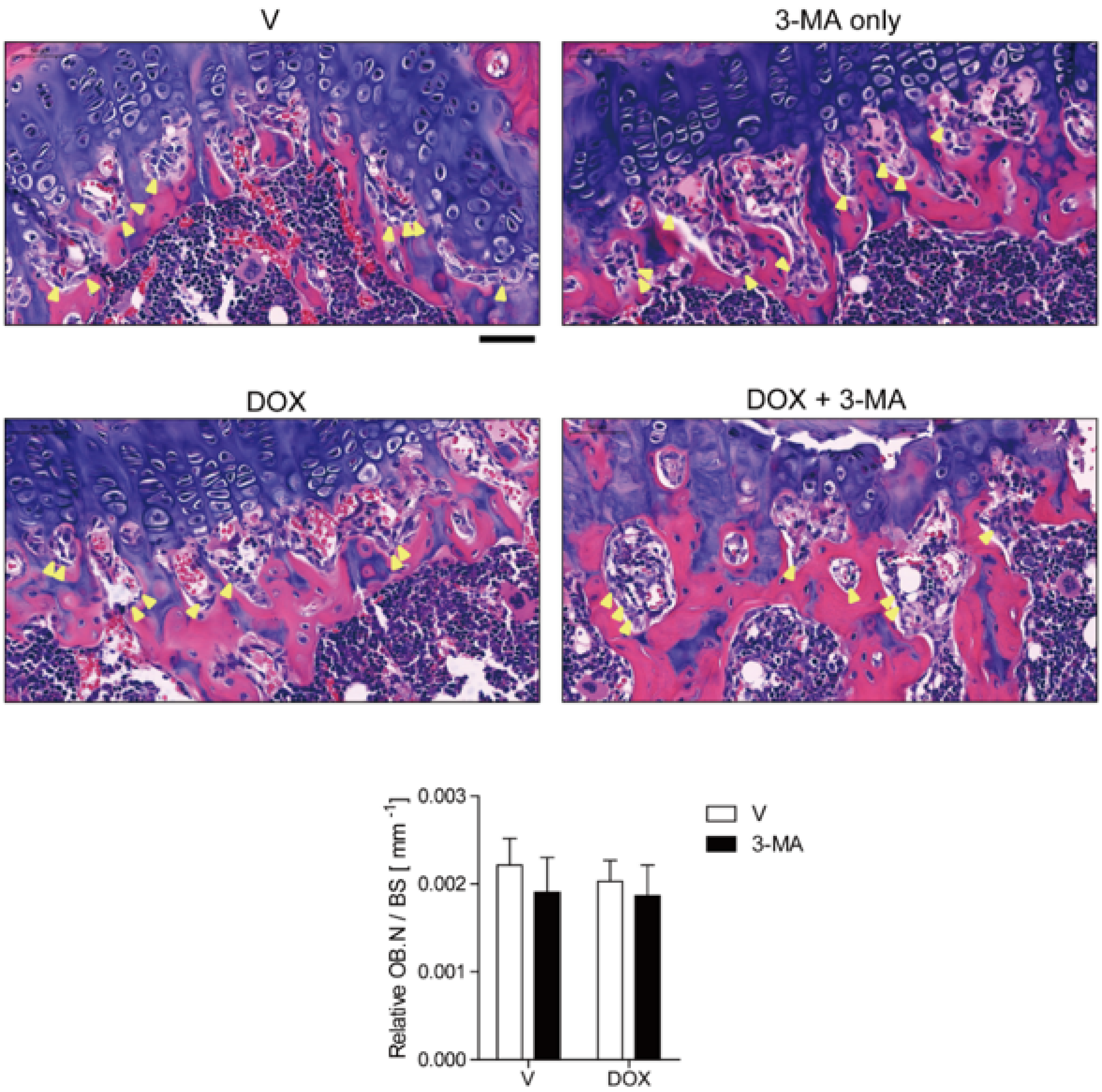

Supplementary Figure S1. Representative images of hematoxylin and eosin (H&E) staining in distal femora from each of the four groups. Osteoblasts (OBs) that have diffused chromatin compared with other cells are indicated by the arrowheads and the relative OB.N/BS ratio (relative OB number divided by total bone surface) was calculated. Scale bar = 50 µm.
